# Chronic conditions and adolescents’ psychosocial wellbeing: the impact of self-reporting

**DOI:** 10.1007/s00431-025-06616-5

**Published:** 2025-12-13

**Authors:** Emma E. Berkelbach van der Sprenkel, Sabine E. I. van der Laan, Catrin Finkenauer, Virissa C. Lenters, Elise M. van de Putte, Louis J. Bont, Cornelis K. van der Ent, Sanne L. Nijhof

**Affiliations:** 1 Department of (Social) Pediatrics, Wilhelmina Children’s Hospital, University Medical Center Utrecht, Utrecht University, Lundlaan 6, 3584 EA Utrecht, the Netherlands; 2 Department of Pediatric Pulmonology, Wilhelmina Children’s Hospital, University Medical Center Utrecht, Utrecht University, Utrecht, the Netherlands; 3 Department of Interdisciplinary Social Sciences, Utrecht University, Utrecht, the Netherlands; 4 Julius Center for Health Sciences and Primary Care, University Medical Center Utrecht, Utrecht, the Netherlands; 5 Department of Environment and Health, Amsterdam Institute for Life and Environment, Vrije Universiteit Amsterdam, Amsterdam, the Netherlands; 6 Department of Infectious Diseases, Wilhelmina Children’s Hospital, University Medical Center Utrecht, Utrecht University, Utrecht, the Netherlands

**Keywords:** Adolescent, Chronic condition, Psychosocial, Wellbeing

## Abstract

This study explores psychosocial wellbeing in adolescents with a physician-diagnosed chronic condition, with emphasis on the disparity between adolescents who self-report their condition (reporters) and those who do not (non-reporters). We also investigated whether these differences varied across disease groups and whether sex, age, and socioeconomic status (SES) moderated associations. This cross-sectional study included participants from the Dutch PROactive cohort aged 12–18 years with a clinical diagnosis of chronic disease: auto-immune disease, cystic fibrosis, congenital heart disease, nephrological condition, or a general pediatric condition. Psychosocial wellbeing was assessed using self-reported indicators of life satisfaction, self-rated health, psychosomatic symptoms, pediatric quality of life, anxiety, and depression. We examined differences in psychosocial wellbeing among reporters and non-reporters, both at a group level and within distinct disease categories. Potential moderating effects of age, sex, and SES on the association between reporting status and psychosocial wellbeing outcomes were assessed. Of the 1009 adolescents (mean age 15.4 ± 1.6 years; 67.4% girls) 26.8% self-reported having a chronic condition. Reporters consistently indicated worse psychosocial wellbeing across all assessed sub-domains. When stratified for disease, this pattern was not always replicated; similar deficits in wellbeing were only observed for the populations with an autoimmune disease or a general pediatric condition. No clear moderating effects were found for sex, age, and SES on the association between self-reporting of chronic disease and psychosocial wellbeing.

*Conclusions*: Only a minority of adolescents with a physician-diagnosed chronic condition self-report as having a chronic disease. Adolescents with a self-reported chronic condition indicated a lower psychosocial wellbeing, a pattern that seems to hold across some, but not all, disease groups.

**Table:** 

**What is Known:** • * Adolescents with chronic conditions are at increased risk of poorer psychosocial wellbeing, particularly girls, older youth, and those with lower SES. While one in four has a physician-diagnosed condition, far fewer self-identify, likely reflecting factors such as disease knowledge, burden, visibility, acceptance, and stigma. How psychosocial wellbeing differs between reporters and non-reporters remains unclear.*	
**What is New:** • * Among adolescents with physician-diagnosed chronic conditions, only a minority self-report as having a chronic disease. Those who do self-report consistently indicate lower psychosocial wellbeing, a pattern which seems to hold in some, but not all, studied disease groups.*

**Supplementary Information:**

The online version contains supplementary material available at 10.1007/s00431-025-06616-5.

## Introduction

Chronic conditions affect a substantial proportion of adolescents worldwide. Based on healthcare records approximately 25% of youth have been clinically diagnosed with at least one chronic somatic or psychiatric condition [1–3]. In contrast, population-representative research revealed that approximately 5% of adolescents aged 11 to 16 years reported having a chronic condition, and these adolescents reported poorer outcomes in a wide range of psychosocial wellbeing domains [3, 4]. This discrepancy illustrates a key challenge in the field: most studies rely exclusively on either self-report or clinical diagnoses, making it difficult to understand how physician-diagnosed and self-reported conditions align and what this means for psychosocial outcomes [1–3, 5].

Youth with a physician-diagnosed chronic condition generally report a significantly lower quality of life, are more prone to develop psychosocial problems, often show delays in achieving psychosocial milestones, and are less likely to be (financially) independent in young adulthood, compared to their healthy peers [2, 5, 6]. Outcomes are not uniform across conditions: adolescents with illnesses that have a variable course or later onset often experience greater psychosocial difficulties [7, 8]. Moreover, previous studies have shown that demographic characteristics such as being female, being older, and having a lower socioeconomic status (SES) have a negative effect on psychosocial wellbeing [7, 9–13].

Many young people—especially those diagnosed early in life—come to regard their condition as part of normal life and do not consider themselves ill, even when medically classified otherwise [8]. Whether adolescents self-report a chronic condition likely depends on factors such as disease knowledge, perceived burden, visibility, acceptance, illness identity, and stigma, and may also reflect self-management, as recognition of illness can precede health-related decisions [14–17]. How adolescents who self-report their condition (“reporters”) differ from those who do not (“non-reporters”) remains unclear, yet this discordance may offer important insights into adolescents’ illness perceptions and their link to psychosocial functioning. In line with Leventhal’s Common-Sense Model of Self-Regulation, adolescents form cognitive and emotional representations of their illness that guide coping and health-related behavior [18]. Self-identifying as chronically ill therefore reflects more than awareness of a diagnosis; it represents how adolescents perceive and relate to their condition, which may in turn influence their coping, self-management, and psychosocial wellbeing. How adolescents integrate their condition into their sense of self– often described as illness identity– may further shape wellbeing [19]. A more integrated illness identity, characterized by acceptance or even enrichment, has been linked to better psychosocial outcomes [19]. Self-identifying as chronically ill may therefore also represent a meaningful indicator of psychosocial adaptation. Thus, distinguishing between reporters and non-reporters could refine our understanding of psychosocial risk and inform more tailored interventions.

In this study we examined psychosocial wellbeing among adolescents with a physician-diagnosed chronic condition, comparing reporters and non-reporters. Secondly, to assess whether the clinical diagnosis played a role, we explored whether differences varied across specific disease groups. Finally, we investigated whether sex, age, and SES—factors previously shown to be associated with poorer psychosocial wellbeing in adolescents with a chronic condition—moderated the associations between self-report of a chronic condition and psychosocial wellbeing [13]. We hypothesized that (1) reporters would show poorer psychosocial wellbeing than non-reporters; (2) these differences would be most pronounced in conditions with a more variable course and later onset; and (3) sex, age, and SES would moderate this association, with female sex, older age, and lower SES linked to poorer psychosocial wellbeing.

## Methods

### Study design and study population

The PROactive cohort study is an ongoing longitudinal study that commenced in 2016 at the Wilhelmina Children’s Hospital, part of the University Medical Center Utrecht, the Netherlands focusing on fatigue, daily life participation, and psychosocial wellbeing [20] [17, 18]. Data are collected from children with a chronic condition. A chronic condition is defined as a clinically established diagnosis with persistent or recurring symptoms lasting more than 3 to 6 months or occurring more than three times per year, requiring long-term use of medications, treatments, or supportive devices [2]. Children were included in the PROactive cohort study if they were aged 2–18 years, at least 1 year post-diagnosis of a chronic condition, or if they presented with complaints of long-lasting fatigue or pain [20]. All questionnaires were available in validated Dutch versions and were self-administered online at home by the adolescents via the secure PROactive KLIK cohort platform. Insufficient proficiency in Dutch to complete the questionnaires was an exclusion criterion [20].

The current study included the cross-sectional baseline data collected from participants aged 12–18 years, resulting in 1009 inclusions. We focused on adolescents aged 12–18 years, as this developmental stage is marked by identity formation, increasing autonomy, and growing responsibility for health management, processes that are likely related to self-reporting of chronic illness [21]. The data refer to the initial set of questionnaires adolescents completed between December 2018 and March 2022.

The PROactive study was classified by the institutional review board as exempt from the Medical Research Involving Human Subjects Act (16–707/C) and adhered to all local laws and the declaration of Helsinki [20]. Digital informed consent was obtained from both the child and their parent(s), covering the use of questionnaire data for research purposes and the extraction of information from the child’s medical records.

### Measures

#### Self-report of a chronic condition

Information about the physician-diagnosed medical diagnosis of each participant was derived from the hospital electronic health record. To assess the presence of self-reported chronic conditions, we used a question from the Dutch Health Behaviour in School-aged Children (HBSC) 2017 questionnaire [22]: “Is there someone in your home (the house or family where you are most of the time) who has been physically and/or mentally ill or disabled for more than 3 months?”, followed by a list of examples: “Examples of diseases and disabilities include: cancer, diabetes, heart disease, depression, addiction, autism, intellectual disability.”. Adolescents could select one or more response options (“yes, myself;” “yes, my father/mother;” “yes, my brother/sister;” or “no, nobody”) to indicate whether they or someone in their household had a chronic condition. Based on this answer, the adolescents were divided into two groups: adolescents with a medical diagnosis who self-reported having a chronic condition (reporters), and adolescents with a medical diagnosis who did not self-report having a chronic condition (non-reporters). We note that this operationalization carries a risk of misclassification, which we address in the limitations.

#### Psychosocial wellbeing

Psychosocial wellbeing was assessed using multiple indicators, including life satisfaction [23, 24], self-rated health [25], psychosomatic health [26], health-related quality of life [27], and symptoms of anxiety and depression [28]. This intentional multidimensional approach aligns with contemporary pediatric health frameworks and Dutch recommendations for generic patient-reported outcome measures (“adviesrapport generieke PROMs”).

Life satisfaction was assessed with the Cantril ladder using the question “How do you feel about your life as a whole right now?” using a ladder analogy and possible answers range from 0 (worst life you can imagine) to 10 (best life you can imagine). However, in the PROactive study, the response options ranged from 1 to 10. The Cantril ladder is considered a valid and reliable instrument for measuring life satisfaction in adolescents [23, 24, 29].

Self-rated health (SRH) was assessed using the HBSC question “What do you think of your own health?” with response categories from 1 (excellent) to 4 (bad) [25]. This construct is widely employed globally and relates to the self-perception of health. It serves as a valid predictor of both mortality and morbidity in adults [30, 31].

Psychosomatic health was assessed using the HBSC Symptom Checklist (HBSC-SCL), and it was expressed as a mean score indicating how frequently specific symptoms were experienced. In total 10 symptoms are assessed (e.g., having a headache, being nervous, or feeling dizzy), and the mean score over these 10 symptoms is calculated [26]. A five-point Likert scale was used to measure the frequency of the symptoms: from 1 (about every day) to 5 (rarely or never). The HBSC-SCL has good psychometric properties and has also been validated in Dutch [26, 32].

Health-related quality of life (QoL) is measured using the Pediatric Quality of Life Generic Core Scale 4.0 (PedsQL GCS), comprising 23 items rated on a 5-point Likert scale. The response options range from 0 (never a problem) to 4 (almost always a problem) [27]. The PedsQL GCS assesses four domains: physical functioning, emotional functioning, social functioning, and school functioning. Answers were reverse coded to create a scale ranging from 0 to 100, where higher scores reflect a higher quality of life. The instrument has been validated for use in Dutch children and adolescents, showing satisfactory psychometric properties with internal consistency coefficients ranging from *α* = 0.53–0.85 across age groups [27]. Reliability is highest for the total scores, whereas lower *α* values (*α* < 0.70) for brief subscales such as school functioning are common and expected given their limited number of items and heterogeneous content [27]. These findings have been replicated in a recent large Dutch population (e.g. [33]), and subscale-level results should therefore be interpreted with caution.

The severity of self-reported anxiety symptoms and depressive symptoms was evaluated using the Revised Child Anxiety and Depression Scale (RCADS), based on anxiety disorders and depression criteria outlined in the Diagnostic and Statistical Manual of Mental Disorders-IV [28]. The questionnaire consists of 47 items, rated on a 4-point Likert scale, ranging from 0 (never) to 3 (always). To assess anxiety symptoms, we used the total anxiety subscale, based on 37 items, with a total score ranging from 0 to 111. To assess depressive symptoms, we used the major depressive disorder subscale, based on 10 items, with a total score ranging from 0 to 30. A higher score indicated more anxiety or depressive symptoms [28]. Raw scores were transformed into normative *T*-scores based on sex and age and assessed as a continuous measure where < 65 = normal, 65–70 = borderline, > 70 = clinical [34]. The RCADS is a reliable and valid instrument, with a good internal consistency (*α* = 0.70–0.96) [35].

Correlations between psychosocial indicators were moderate to strong (Supplement 1), indicating related but non-redundant domains.

#### Individual characteristics

Age, sex, and disease group were obtained from the electronic medical record and included as individual characteristics. The disease groups were auto-immune disease, cystic fibrosis (CF), congenital heart disease (CHD), nephrological conditions, and general pediatric conditions (for details on the included conditions, please see [20]). The general pediatric conditions predominantly included persistent somatic symptoms (e.g., chronic pain or fatigue), and to a lesser extent asthma, obesity, and mood or anxiety disorders. To enhance transparency, we provide a full list of included “general pediatric” diagnoses in Supplement 3.

SES was also included as an individual characteristic and calculated using the SES-score from Statistics Netherlands, which is based on the financial prosperity, educational level, and recent employment history of residents of a neighborhood administrative unit (four-digit postal code) [36]. The postal code of each participant was retrieved from the electronic medical record. The SES-score is freely available and ranges from − 0.89 to 0.76 for Dutch households, with a higher coefficient indicating higher SES [37].

### Statistical analysis

We used descriptive statistics to summarize the characteristics of the study population– for the total population, and separately for the reporters and the non-reporters. To evaluate demographic differences between reporters and non-reporters, we performed χ2 tests for categorical data, and for continuous data we used independent t-tests when the data were normally distributed or Mann–Whitney* U* tests for skewed distributed data. Spearman’s rank correlation coefficients were used to examine the correlations between different psychosocial wellbeing measures (Supplement 1).

To assess differences in psychosocial wellbeing outcomes between reporters and non-reporters, we used the same tests described above. To gain more insight into the relationship between reporting status and psychosocial wellbeing in specific types of chronic conditions, we performed these analyses stratified by disease group. Secondary analyses were conducted to explore the potential moderation effects of the associations between reporting status and psychosocial wellbeing outcomes by age, sex, and SES. We used generalized linear models with psychosocial wellbeing as the dependent variable (each outcome analyzed separately), and two-way interaction terms between reporting (yes, no) and potential moderators as the independent variables. *Z*-scores were employed for the continuous variables (age and SES) to facilitate standardized comparisons. No further covariates were included. Model assumptions were checked and found to be met.

We presented the beta with 95% confidence intervals for the main effects of reporting status, age, sex, and SES. Significance levels were set at *α* < 0.05. For each psychosocial wellbeing outcome domain, four tests were conducted: one primary analysis, and three secondary analyses testing potential moderator effects. To account for multiple comparisons within each outcome domain, a post hoc Bonferroni correction was applied. Consequently, for our outcomes, results with a *p*-value of 0.0125 or lower were considered statistically significant. All analyses were performed with SPSS 27.0 (IBM, Armonk, NY, USA).

## Results

In total, 1009 adolescents were included, with a mean age of 15.4 years (*σ* = 1.6), of which 67.4% were girls. Out of the total sample, 57.0% were patients with a general pediatric condition, 26.4% had an auto-immune disease, 9.0% had a CHD, 5.1% had CF, and 2.7% had a nephrological condition. Of the patients with a general pediatric condition, 89.5% of 574 adolescents experienced persistent somatic symptoms (mostly pain or fatigue). Of these, 59.8% also had another chronic condition. The remaining 10.5% without persistent somatic symptoms had another chronic condition, such as asthma, obesity, or a mood disorder. Please refer to Supplement 3 for additional details on the patients with a general pediatric condition.

### Reporting status

In general, 26.8% (*n* = 270) reported having a chronic condition (Table 1). Individual characteristics such as age, sex, disease group, and SES did not significantly differ between reporters and non-reporters (Table 1). Among specific disease groups, the percentage of reporters ranged from 23% (CHD) to 44% (nephrological conditions).

**Table 1:** Baseline characteristics for study population and stratified by reporting status

Individual characteristics		Total *n* = 1009		Reporters *n* = 270		Non-reporters *n* = 739		*p*-value
Age at baseline	*n* = 1009	15.4	± 1.6	15.5	± 1.6	15.3	± 1.6	0.16
Sex	*n* = 1002							0.40
Male		322	(32.1)	92	(34.2)	230	(31.4)	
Female		680	(67.9)	177	(65.8)	503	(68.6)	
Disease group	*n* = 1009							0.29
Auto-immune		266	(26.4)	72	(26.7)	194	(26.3)	
Cystic fibrosis		51	(5.1)	14	(5.2)	37	(5.0)	
Congenital heart disease		91	(9.0)	21	(7.8)	70	(9.5)	
General pediatric conditions		574	(57.0)	151	(55.9)	423	(57.2)	
Nephrological conditions		27	(2.7)	12	(4.4)	15	(2.0)	
Socioeconomic status^1^	*n* = 975	0.1	± 0.2	0.1	± 0.2	0.1	± 0.2	0.49

Data are presented as *n*, mean ± standard deviation or *n* (%). ^1^Range from − 0.89 to 0.76 for Dutch households [37]

### Differences in psychosocial wellbeing between reporters and non-reporters

Reporters demonstrated lower scores on all separate psychosocial wellbeing outcomes (*p-*values ranging from < 0.001 to 0.009) compared to non-reporters (Table 2). To be more specific, reporters reported significantly lower life satisfaction (*M* = 6.3 vs. 7.0, *d* = 0.4, 95% CI [0.2–0.5], *p* < 0.001), poorer self-rated health (*M* = 3.1 vs. 2.8, *d* = − 0.4, 95% CI [− 0.6 to − 0.3],* p* < 0.001), and lower levels of psychosomatic health (*M* = 3.1 vs. 3.3, *d* = 0.3, 95% CI [0.2–0.6], *p* < 0.001). Health-related QoL was also lower among reporters compared to non-reporters (total score 62.8 vs. 70.5, *d* = 0.5, 95% CI [0.3–0.6], *p* < 0.001), with consistent differences across subscales (for details, refer to Table 2). Finally, reporters reported more anxiety (*M* = 42.6 vs. 40.2, *r* = 0.1, *p* = 0.009) and depressive symptoms (*M* = 54.4 vs. 49.8, *r* = 0.2, *p* < 0.001).

**Table 2:** Differences in psychosocial wellbeing between reporters and non-reporters

Psychosocial wellbeing outcomes		Total *n* = 1009		Reporters *n* = 270		Non-reporters *n* = 739		Effect size	(95% CI)	*p*-value
Life satisfaction [range 1–10]	*n* = 1009	6.8	± 1.8	6.3	± 1.9	7.0	± 1.7	0.4^a^	(0.2;0.5)	< 0.001
Self-rated health [range 1–4]	*n* = 1009	2.9	± 1.6	3.1	± 0.8	2.8	± 0.8	− 0.4^a^	(− 0.6; − 0.3)	< 0.001
Psychosomatic health [range 1–5]	*n* = 1009	3.3	± 0.9	3.1	± 0.9	3.3	± 0.9	0.3^a^	(0.2;0.5)	< 0.001
Health-related quality of life [range 1–100]	*n* = 802	68.5	± 17.2	62.8	± 18.4	70.5	± 16.3	0.5^a^	(0.3;0.6)	< 0.001
Physical		66.6	± 23.5	59.2	± 26.0	69.1	± 22.1	− 0.2^b^	N/A	< 0.001
Emotional		68.8	± 21.1	64.6	± 21.1	70.3	± 20.9	− 0.1^b^	N/A	0.001
Social		79.7	± 17.0	74.2	± 19.6	81.6	± 15.6	− 0.2^b^	N/A	< 0.001
School		60.0	± 21.0	55.3	± 21.4	61.7	± 20.7	0.3^a^	(0.15;0.46)	< 0.001
Internalizing symptoms [*t*-score]^1^	*n* = 932									
Anxiety symptoms		40.8	± 10.5	42.6	± 11.8	40.2	± 9.9	0.1^b^	N/A	0.009
Depressive symptoms		51.1	± 13.4	54.4	± 13.9	49.8	± 13.0	0.2^b^	N/A	< 0.001

Data are presented as *n* or mean ± standard deviation. N/A means not applicable. ^1^Raw scores were converted to normative *T*-scores based on sex and age, where < 65 = normal, 65–70 = borderline, > 70 = clinical. ^a^ = Cohen’s *d*; ^b^ = *r*

### Relation between reporting status and psychosocial wellbeing across chronic conditions

When stratifying the analysis based on disease groups, similar results were observed among adolescents with an auto-immune disorder and a general pediatric condition; reporters exhibited a lower psychosocial wellbeing in comparison to non-reporters (Supplement 2). Among adolescents with an auto-immune disease (*n* = 266), reporters showed lower life satisfaction, poorer self-rated health, worse psychosomatic health, lower health-related QoL with decrements across all subscales (*d* = 0.3 to 0.6, *p* = < 0.001–0.02,) and more anxiety and depressive symptoms (*r* = 0.2,* p* = 0.001–0.004). Among adolescents with a general pediatric condition (*n* = 574), reporters indicated lower life satisfaction, poorer self-rated health, worse psychosomatic health, lower health-related QoL with decrements across all subscales except emotional functioning (*d* = 0.3–0.7, *p* ≤ 0.001), and more depressive symptoms (*r* = 0.2, *p* < 0.001). No significant differences were found in the psychosocial wellbeing of reporters compared to non-reporters for adolescents with CF, CHD, and nephrological conditions. Because of the heterogeneity within disease groups and the relatively small sample sizes in CF, CHD, and nephrological conditions (*n* = 27–91), these stratified analyses should be interpreted with caution and are provided mainly as additional context.

### Moderating effects of sex, age, and SES on the association between reporting status and psychosocial wellbeing

The analyses exploring the moderating effects of demographic factors included main effects and interactions with moderators. Replicating the results of the direct comparisons, the main effects of reporters were negatively associated with psychosocial wellbeing, meaning that reporters had worse psychosocial wellbeing compared to non-reporters (Table 3). There was one exception: being a reporter was not associated with a higher degree of anxiety (*β* = 1.96, 95% CI [0.14–3.77], *p* = 0.03), when the moderating effect of sex on reporting status and anxiety symptoms was tested. The main effect of sex was significant for all outcomes. Girls were more likely to experience lower life satisfaction (*β* = 0.64, 95% CI [0.37 to 0.90], *p* < 0.001), poorer self-rated health (*β* = –0.38, 95% CI [–0.51 to –0.25], *p* < 0.001), worse psychosomatic health (*β* = 0.63, 95% CI [0.49 to 0.76], *p* < 0.001), and lower health-related QoL (*β* = 9.68, 95% CI [6.87 to 12.48], *p* < 0.001), as well as more anxiety (*β* = − 4.37, 95% CI [− 6.05 to − 2.70], *p* < 0.001) and depressive symptoms (*β* = − 6.25, 95% CI [− 8.36 to 4.14], *p* < 0.001). The main effect of age was only significant for the outcomes of life satisfaction (*β* = –0.30, 95% CI [–0.43 to –0.18], *p* < 0.001), self-rated health (*β* = 0.15, 95% CI [0.09–0.21], *p* < 0.001), and psychosomatic health (*β* = –0.11, 95% CI [–0.17 to –0.05], *p* = 0.001), indicating that older adolescents had worse outcomes in these domains. No main effect for SES was found across all psychosocial wellbeing outcomes. In general, no moderating effects for sex, age, and SES were found. We found indications of an interaction between sex and reporting status on psychosomatic health (*β* = –0.28, 95% CI [–0.54 to –0.03], *p* = 0.03) and on depressive symptoms (*β* = 4.13, 95% CI [0.15–8.12], *p* = 0.04), although those results did not survive correction for multiple comparisons.

**Table 3:** Moderating effects of sex, age and SES on the association between reporting status and psychosocial wellbeing

Sex					Age					SES				
**Variables**	***β***	**SE**	**95% CI**	***p***	**Variables**	***β***	**SE**	**95% CI**	***p***	**Variables**	***β***	**SE**	**95% CI**	***p***
**Life satisfaction**^**1**^														
Reporter	− 0.65	0.15	(− 0.94; − 0.35)	< 0.001	Reporter	− 0.64	0.12	(− 0.87; − 0.40)	< 0.001	Reporter	− 0.68	0.12	(− 0.92; − 0.43)	< 0.001
Sex*	0.64	0.08	(0.37; 0.90)	< 0.001	Age●	− 0.30	0.06	(− 0.43; − 0.18)	< 0.001	SES●	− 0.01	0.06	(− 0.14; 0.12)	0.87
Interaction	− 0.11	0.26	(− 0.62; 0.39)	0.66	Interaction	0.06	0.12	(− 0.18; 0.30)	0.66	Interaction	0.09	0.12	(− 0.16; 0.33)	0.49
**Self-rated health**^**2**^														
Reporter	0.37	0.07	(0.22; 0.51)	< 0.001	Reporter	0.36	0.06	(0.24; 0.47)	< 0.001	Reporter	0.39	0.06	(0.27; 0.50)	< 0.001
Sex*	− 0.38	0.07	(− 0.51; − 0.25)	< 0.001	Age●	0.15	0.03	(0.09; 0.21)	< 0.001	SES●	− 0.006	0.03	(− 0.07; 0.06)	0.85
Interaction	0.04	0.12	(− 0.20; 0.29)	0.74	Interaction	0.02	0.06	(− 0.09; 0.14)	0.71	Interaction	0.02	0.06	(− 0.10; 0.13)	0.81
**Psychosomatic health**^**3**^														
Reporter	− 0.21	0.07	(− 0.36; − 0.06)	0.005	Reporter	− 0.27	0.06	(− 0.40; − 0.15)	< 0.001	Reporter	− 0.30	0.06	(− 0.43; − 0.17)	< 0.001
Sex*	0.63	0.07	(0.49; 0.76)	< 0.001	Age●	− 0.11	0.03	(− 0.17; − 0.05)	0.001	SES●	0.009	0.03	(− 0.06; 0.07)	0.79
Interaction	− 0.28	0.13	(− 0.54; − 0.03)	0.03	Interaction	0.02	0.06	(− 0.10; 0.15)	0.73	Interaction	0.02	0.06	(− 0.10; 0.15)	0.71
**Health-related QoL**^**4**^														
Reporter	− 7.06	1.65	(− 10.29; − 3.83)	< 0.001	Reporter	− 7.62	1.36	(− 10.28; − 4.95)	< 0.001	Reporter	− 7.59	1.38	(− 10.30; − 4.88)	< 0.001
Sex*	9.68	1.43	(6.87; 12.48)	< 0.001	Age●	− 0.69	0.67	(− 2.01; 0.64)	0.31	SES●	− 0.16	0.71	(− 1.54; 1.22)	0.82
Interaction	− 2.45	2.77	(− 7.89; 2.99)	0.38	Interaction	− 0.31	1.35	(− 2.96; 2.34)	0.82	Interaction	1.32	1.40	(− 1.43; 4.07)	0.35
**Anxiety symptoms**^**5**^														
Reporter	1.96	0.93	(0.14; 3.77)	0.03	Reporter	2.38	0.77	(0.86; 3.89)	0.002	Reporter	2.42	0.78	(0.89; 3.94)	0.002
Sex*	− 4.37	0.85	(− 6.05; − 2.70)	< 0.001	Age●	0.22	0.40	(− 0.57; 1.01)	0.59	SES●	0.76	0.41	(− 0.04; 1.55)	0.06
Interaction	1.63	1.61	(− 1.53; 4.78)	0.31	Interaction	− 0.08	0.78	(− 1.61; 1.45)	0.92	Interaction	− 0.01	0.77	(− 1.52; 1.50)	0.99
**Depressive symptoms**^**6**^														
Reporter	3.28	1.17	(0.98; 5.57)	0.005	Reporter	4.49	0.98	(2.57; 6.40)	< 0.001	Reporter	4.69	0.99	(2.75; 6.62)	< 0.001
Sex*	− 6.25	1.08	(− 8.36; − 4.14)	< 0.001	Age●	0.10	0.51	(− 0.90; 1.10)	0.85	SES●	0.57	0.51	(− 0.43; 1.58)	0.27
Interaction	4.13	2.03	(0.15; 8.12)	0.04	Interaction	0.16	0.99	(− 1.78; 2.10)	0.87	Interaction	− 0.75	0.98	(− 2.66; 1.16)	0.44

*β*, unstandardized *β*; *95% CI*, 95% confidence intervals; *SE*, standard error; *SES*, socioeconomic status. *Estimate for male, reference is female. ●*Z*-score is used; when this *Z*-score is used in the independent variable, it is also used in the interaction term. ^1^Higher score indicates better life satisfaction; ^2^Lower score indicates better self-rated health; ^3^Higher score indicates better psychosomatic health; ^4^Higher score indicates better quality of life; ^5^Higher score indicates more anxiety symptoms; ^6^Higher score indicates more depressive symptoms

## Discussion

The present study aimed to investigate differences in psychosocial wellbeing among adolescents with a physician-diagnosed chronic condition who self-report or do not self-report having a chronic condition. Out of the 1009 included adolescents with a clinical diagnosis, 270 (26.7%) were reporters. Our findings revealed that reporters had significantly worse outcomes in all psychosocial domains assessed. When stratified by disease group, reporters with general pediatric conditions or with an auto-immune disease indicated a significantly lower psychosocial wellbeing than non-reporters. No significant differences between reporters and non-reporters were found for the adolescents with CF, a CHD, and a nephrological condition (detailed results are provided in the Supplementary Tables). In general, no clear moderating effects of sex, age, or SES on the relationship between reporting status and psychosocial wellbeing, were identified. Several observed differences were clinically meaningful. For instance, the 6–7-point lower PedsQL scores among reporters exceed the minimal clinically important difference (MCID) of 4.5 points, indicating a decrement likely to be noticeable in daily functioning [38]. Differences in life satisfaction, psychosomatic health, and self-rated health may also be relevant for adolescents’ wellbeing and participation, whereas the effects on anxiety and depression (RCADS) were small and below clinical thresholds [34]. Taken together, these results suggest that self-report status is informative not only statistically, but also clinically.

Discrepancies between medical and self-reported chronic conditions have also been observed in population-based studies. For example, the German KiGGS cohort, using the *Children with Special Health Care Needs* (CSHCN) Screener, found that self-reported chronic illness—particularly among adolescents with pain, asthma, or lower socioeconomic status—predicted later self-identification and greater healthcare use [39, 40]. Whereas the CSHCN focuses on functional limitations and care needs, our HBSC-based item captures adolescents’ subjective perception of having a chronic condition. Together, these approaches highlight that self-reporting reflects both functional burden and illness perception, and that adolescents’ self-identification may evolve over time [39].

This study confirmed that reporting is associated with impaired psychosocial wellbeing, consistent with a previous population study, while also extending the findings by incorporating medical data from the study population [3, 13]. Interestingly, the majority (73.2%) of adolescents with a physician-diagnosed chronic condition did not self-report having a chronic condition. The percentage of self-reporters varied among disease groups, ranging from 23% (CHD) to 44% (nephrological conditions). Several hypotheses can be proposed regarding factors that might influence reporting status. First, reporters may experience more severe disease symptoms. Moreover, the extent to which one reports having a chronic condition might be associated with the degree of perceived burden, irrespective of the disease severity. Furthermore, environmental factors, including parents and friends, can influence adolescents’ self-reporting of chronic conditions. Factors such as disease knowledge, awareness, conversations about the illness, and social stigma or acceptance within one’s social circles have been found significant in the context of self-report [14–16]. Lastly, illness identity—how individuals construct or reestablish a new sense of self, wherein the condition becomes integrated into their identity—might also have influenced the decision to report having a chronic condition [41, 42]. Adolescents who perceive their illness as more central or burdensome may be more likely to self-report it and experience lower psychosocial wellbeing, whereas those who have normalized or accepted their condition as part of everyday life may be less likely to self-identify as chronically ill. Considering illness identity as part of the self-reporting process may therefore help explain the observed differences in psychosocial outcomes [19]. The PROactive cohort structure with preset questionnaires limits detailed examination of these specific concepts related to self-report, marking this study as an initial exploration and emphasizing the need for more comprehensive research.

The negative correlation between reporting and psychosocial wellbeing prompts inquiry. The hypotheses regarding self-report of a chronic condition could also be relevant here. Factors such as increased disease severity, higher perceived burden, heightened stigmatization, and negative illness identities may contribute to lower psychosocial wellbeing. It is, for instance, recognized that individuals who share similar experiences may interpret or evaluate those experiences differently [43, 44]. The level of *perceived* burden influences subsequent stress responses [45, 46]. Furthermore, psychosocial wellbeing and reporting status could also be related to how adolescents evaluate their overall health. In a previous study, children defined health as “feeling good about yourself” and “being able to participate.” Through interviews, six domains of health were identified, each with various related aspects: body, feelings and thoughts, now and in the future, feeling good about yourself, participation, and daily life [47]. This aligns with the contemporary societal perspective on health, characterized by a shift from a disease-centric perspective to a broader, more all-encompassing approach to health with an increased emphasis on self-management and the ability to adapt [48]. It is conceivable that non-reporting in our study reflects perceived control over other health domains irrespective of the condition and is thus also related to better psychosocial wellbeing overall. Clinically, our findings suggest that adolescents who self-identify as having a chronic condition may represent a group at higher risk of poorer psychosocial wellbeing. Addressing this theme in routine care and paying attention to the reasons adolescents perceive themselves this way may provide a practical approach to identify more vulnerable patients and adapt (psychosocial) support to their needs.

To better understand the relationship between the medical diagnosis of a chronic condition, reporting status, and psychosocial wellbeing, future research should prioritize more targeted designs rather than larger samples. Longitudinal studies could track trajectories of self-reporting across adolescence and examine their relationship with disease-specific factors such as perceived burden, while qualitative work may shed light on processes such as illness identity, disclosure, and stigma. Mixed-methods approaches that combine quantitative outcomes with adolescents’ lived experiences may be especially valuable in capturing this complexity. Enhancing our understanding of adolescents’ reporting behavior and psychosocial wellbeing has significance for improving care. It will contribute to shifting healthcare professionals’ focus towards a more patient-centered model of care with a broader perspective on health, moving beyond traditional disease-centered paradigms.

In the present study, we evaluated a comprehensive sample drawn from a large academic pediatric hospital in the Netherlands, encompassing adolescents with diverse disease types. Owing to the known medical information, we were able to further illuminate the previously delineated discrepancies between the prevalence of physician-diagnosed and self-reported chronic conditions. Several limitations of this study warrant acknowledgment. First, the cross-sectional design precludes conclusions about causality or the temporal direction between reporting status and psychosocial wellbeing. Second, the stratified analyses should be interpreted with caution due to the relatively small sample sizes and heterogeneity within some disease groups, particularly the “general pediatric conditions,” which likely reduced statistical power and interpretability. Additionally, the representativeness of the PROactive cohort may be limited due to the recruitment of patients from an academic hospital, characterized by specific diagnoses, post-establishment of chronic disease diagnosis, and a relatively elevated SES in contrast to the broader population. We also note that our self-report variable was based on a household-level HBSC item, which may be variably interpreted. Misclassification is possible in adolescents whose symptoms were in remission, whose condition was less visible, or who preferred not to self-identify as ill. Besides that, the proportion of patients with a general pediatric condition, a heterogeneous group, adds complexity to our findings. Although persistent somatic symptoms predominated, the presence of comorbidities adds complexity and limits the precision and interpretability of subgroup comparisons. However, the diversity in disease types, age, and sex within the sample may mitigate this limitation to some extent. Lastly, the neighborhood-based SES measure might not fully reflect individual socio-economic nuances.

## Conclusion

This study revealed that only a minority of adolescents with a physician-diagnosed chronic condition self-report as having a chronic disease. Reporters indicated a lower psychosocial wellbeing, a pattern that seems to hold across some, but not all, disease groups. The findings of this study underscore the need to adopt a multidimensional healthcare perspective that emphasizes the transition from a disease-centric to a more individual-centric approach.

## Supplementary Information

Below is the link to the electronic supplementary material.

ESM1 (DOCX 22.2 KB)

ESM2 (DOCX 28.8 KB)

ESM3 (DOCX 112 KB)

## Data Availability

The datasets used and/or analyzed during the current study are available from the corresponding author on reasonable request.The datasets used and/or analyzed during the current study are available from the corresponding author on reasonable request.

## Abbreviations

CF: Cystic fibrosis
CHD: Congenital heart disease
95% CI: 95% Confidence intervals
HBSC: Health Behaviour in School-aged Children
HBSC-SCL: Health Behavior in School-aged Children Symptom Checklist
PedsQL GCS: Pediatric Quality of Life Generic Core Scale 4.0
QoL: Quality of life
RCADS: Revised child anxiety and depression scale
SE: Standard error
SES: Socioeconomic status
SRH: Self-rated health
