# Supplementary material for: Chronic conditions and adolescents’ psychosocial wellbeing: the impact of self-reporting
Source: Eur J Pediatr. 2025 Dec 13;185(1):15. doi: 10.1007/s00431-025-06616-5 (PMC12701850; doi:10.1007/s00431-025-06616-5)
Supplement: Supplementary file 1 — (DOCX 22.2 KB) [file 431_2025_6616_MOESM1_ESM.docx]

**Supplement 1.** Correlations between the domains of psychosocial wellbeing

|  | | | | | | | | | | | |
| --- | --- | --- | --- | --- | --- | --- | --- | --- | --- | --- | --- |
|  |  | PedsQL,  total score | PedsQL,  subscale physical funct. | PedsQL,  subscale emotional funct. | PedsQL,  subscale social funct. | PedsQL,  subscale school funct. | RCADS,  depression score | RCADS,  total anxiety score | Cantril ladder,  life satisfaction | Self-rated health | HBSC-SCL, Psychosomatic health |
| PedsQL,  total score | Spearman’s rho | 1 |  |  |  |  |  |  |  |  |  |
|  | Sig. (2-tailed) |  |  |  |  |  |  |  |  |  |  |
|  | n | 802 |  |  |  |  |  |  |  |  |  |
| PedsQL,  subscale physical funct. | Spearman’s rho | .89 | 1 |  |  |  |  |  |  |  |  |
|  | Sig. (2-tailed) | <0.001 |  |  |  |  |  |  |  |  |  |
|  | n | 802 | 802 |  |  |  |  |  |  |  |  |
| PedsQL,  subscale emotional funct. | Spearman’s rho | .75 | .50 | 1 |  |  |  |  |  |  |  |
|  | Sig. (2-tailed) | <0.001 | <0.001 |  |  |  |  |  |  |  |  |
|  | n | 802 | 802 | 802 |  |  |  |  |  |  |  |
| PedsQL,  subscale social funct. | Spearman’s rho | .78 | .63 | .52 | 1 |  |  |  |  |  |  |
|  | Sig. (2-tailed) | <0.001 | <0.001 | <0.001 |  |  |  |  |  |  |  |
|  | n | 802 | 802 | 802 | 802 |  |  |  |  |  |  |
| PedsQL,  subscale school funct. | Spearman’s rho | .78 | .56 | .56 | .51 | 1 |  |  |  |  |  |
|  | Sig. (2-tailed) | <0.001 | <0.001 | <0.001 | <0.001 |  |  |  |  |  |  |
|  | n | 802 | 802 | 802 | 802 | 802 |  |  |  |  |  |
| RCADS,  the major depressive disorder score | Spearman’s rho | -.77 | -.61 | -.74 | -.55 | -.66 | 1 |  |  |  |  |
|  | Sig. (2-tailed) | <0.001 | <0.001 | <0.001 | <0.001 | <0.001 |  |  |  |  |  |
|  | n | 755 | 755 | 755 | 755 | 755 | 932 |  |  |  |  |
| RCADS,  total anxiety score | Spearman’s rho | -.50 | -.30 | -.68 | -.39 | -.41 | .68 | 1 |  |  |  |
|  | Sig. (2-tailed) | <0.001 | <0.001 | <0.001 | <0.001 | <0.001 | <0.001 |  |  |  |  |
|  | n | 755 | 755 | 755 | 755 | 755 | 932 | 932 |  |  |  |
| Cantril ladder,  life satisfaction | Spearman’s rho | .59 | .47 | .54 | .48 | .48 | -.62 | -.39 | 1 |  |  |
|  | Sig. (2-tailed) | <0.001 | <0.001 | <0.001 | <0.001 | <0.001 | <0.001 | <0.001 |  |  |  |
|  | n | 802 | 802 | 802 | 802 | 802 | 938 | 938 | 1009 |  |  |
| Self-rated  health | Spearman’s rho | -.67 | -.65 | -.43 | -.50 | -.54 | .57 | .27 | -.55 | 1 |  |
|  | Sig. (2-tailed) | <0.001 | <0.001 | <0.001 | <0.001 | <0.001 | <0.001 | <0.001 | <0.001 |  |  |
|  | n | 802 | 802 | 802 | 802 | 802 | 938 | 938 | 1009 | 1009 |  |
| HBSC-SCL,  Psychosomatic health | Spearman’s rho | .72 | .59 | .68 | .46 | .63 | -.79 | -.48 | .56 | -.56 | 1 |
|  | Sig. (2-tailed) | <0.001 | <0.001 | <0.001 | <0.001 | <0.001 | <0.001 | <0.001 | <0.001 | <0.001 |  |
|  | n | 802 | 802 | 802 | 802 | 802 | 938 | 938 | 1009 | 1009 | 1009 |
| Funct.=functioning; PedsQL = Pediatric Quality of Life Inventory; Sig.=significance; RCADS = Revised Child Anxiety and Depression Scale; HBSC-SCL = Health Behaviour in School-aged Children Symptom Checklist.    Using the Bonferroni correction, a significance threshold of *p* < 0.005 was applied. | | | | | | | | | | | |
