# Supplementary material for: Chronic conditions and adolescents’ psychosocial wellbeing: the impact of self-reporting
Source: Eur J Pediatr. 2025 Dec 13;185(1):15. doi: 10.1007/s00431-025-06616-5 (PMC12701850; doi:10.1007/s00431-025-06616-5)
Supplement: Supplementary file 2 — (DOCX 28.8 KB) [file 431_2025_6616_MOESM2_ESM.docx]

**Supplement 2.** Psychosocial functioning per disease group, stratified by identification status

| **Auto-immune disease** | **Total**  ***n*=266** | | **Reporters**  ***n*=72** | | **Non-reporters**  ***n*=194** | | **Effect size** | **(95% CI)** | ***p*-value** |
| --- | --- | --- | --- | --- | --- | --- | --- | --- | --- |
| Life satisfaction [range 1-10] | 7.3 | ±1.6 | 7.0 | ±1.7 | 7.5 | ±1.5 | 0.3 ^a^ | (0.1;0.6) | 0.02 |
| Self-rated health [range 1-4] | 2.5 | ±0.8 | 2.8 | ±0.8 | 2.4 | ±0.8 | -0.5 ^a^ | (-0.8;-0.2) | <0.001 |
| Psychosomatic health [range 1-5] | 3.7 | ±0.9 | 3.4 | ±1.0 | 3.9 | ±0.8 | 0.6 ^a^ | (0.3;0.9) | <0.001 |
| Health-related QoL, total [range 1-100]  Physical  Emotional  Social  School | 76.6  76.3  75.8  84.9  69.6 | ±15.6  ±20.4  ±20.0  ±16.2  ±18.9 | 71.2  71.1  69.2  80.0  64.6 | ±17.4  ±20.4  ±23.0  ±19.5  ±20.8 | 78.4  78.1  78.0  86.5  71.2 | ±14.6  ±20.2  ±18.5  ±14.7  ±18.0 | 0.5 ^a^  -0.2 ^b^  -0.2 ^b^  -0.1 ^b^  0.4 ^a^ | (0.2;0.8)  N/A  N/A  N/A  (0.1;0.7) | 0.002  0.01  0.01  0.03  0.02 |
| Internalizing symptoms [t-score] ^1^  Anxiety symptoms  Depressive symptoms | 38.9  45.0 | ±10.3  ±12.3 | 43.2  50.4 | ±13.8  ±16.1 | 37.3  42.8 | ±8.0  ±9.7 | 0.2 ^b^  0.2 ^b^ | N/A  N/A | 0.004  0.001 |
| **Cystic fibrosis** | **Total**  ***n*=51** | | **Reporters**  ***n*=14** | | **Non-reporters**  ***n*=37** | | **Effect size** | **(95% CI)** | ***p*-value** |
| Life satisfaction [range 1-10] | 7.4 | ±1.6 | 7.5 | ±1.3 | 7.4 | ±1.7 | -0.1 ^a^ | (-0.7;0.5) | 0.81 |
| Self-rated health [range 1-4] | 2.5 | ±0.8 | 2.3 | ±0.8 | 2.5 | ±0.8 | 0.2 ^a^ | (-0.4;0.8) | 0.54 |
| Psychosomatic health [range 1-5] | 3.9 | ±0.7 | 3.6 | ±0.7 | 4.0 | ±0.7 | 0.5 ^a^ | (-0.1;1.1) | 0.12 |
| Health-related QoL, total [range 1-100]  Physical  Emotional  Social  School | 77.0  78.6  74.8  86.7  67.0 | ±15.3  ±19.4  ±20.5  ±14.2  ±17.0 | 76.8  80.1  72.7  89.2  63.5 | ±15.6  ±18.7  ±21.4  ±15.9  ±17.6 | 77.1  78.1  75.6  85.8  68.8 | ±15.4  ±19.8  ±20.4  ±13.7  ±16.8 | <0.1 ^a^  <0.1 ^b^  <-0.1 ^b^  0.2 ^b^  0.3 ^a^ | (-0.6;0.7)  N/A  N/A  N/A  (-0.4;0.9) | 0.96  0.78  0.59  0.25  0.38 |
| Internalizing symptoms [t-score] ^1^  Anxiety symptoms  Depressive symptoms | 38.2  44.9 | ±9.2  ±10.7 | 41.2  47.1 | ±9.9  ±9.4 | 36.9  44.0 | ±8.7  ±11.2 | 0.2 ^b^  0.1 ^b^ | N/A  N/A | 0.18  0.27 |
| **Congenital heart disease** | **Total**  ***n*=91** | | **Reporters**  ***n*=21** | | **Non-reporters**  ***n*=70** | | **Effect sizes** | **(95% CI)** | ***p*-value** |
| Life satisfaction [range 1-10] | 7.4 | ±1.4 | 7.1 | ±1.0 | 7.5 | ±1.4 | 0.2 ^a^ | (-0.3;07) | 0.34 |
| Self-rated health [range 1-4] | 2.2 | ±0.7 | 2.4 | ±0.6 | 2.1 | ±0.7 | -0.3 ^a^ | (-0.8;0.1) | 0.17 |
| Psychosomatic health [range 1-5] | 3.8 | ±0.8 | 3.6 | ±0.9 | 3.9 | ±0.7 | 0.4 ^a^ | (-0.1;0.9) | 0.14 |
| Health-related QoL, total [range 1-100]  Physical  Emotional  Social  School | 77.0  77.8  74.9  82.5  72.3 | ±12.7  ±19.8  ±17.4  ±16.0  ±16.4 | 70.4  67.3  72.7  75.4  68.1 | ±15.1  ±24.6  ±16.8  ±18.7  ±12.2 | 78.7  80.5  75.5  84.3  73.4 | ±11.5  ±17.7  ±17.6  ±14.9  ±17.5 | 0.7 ^a^  -0.2 ^b^  -0.1 ^b^  -0.2 ^b^  0.3 ^a^ | (0.1;1.3)  N/A  N/A  N/A  (-0.3;0.9) | 0.03  0.06  0.58  0.11  0.30 |
| Internalizing symptoms [t-score] ^1^  Anxiety symptoms  Depressive symptoms | 40.0  44.2 | ±9.0  ±11.1 | 40.8  45.4 | ±8.9  ±10.6 | 39.8  43.8 | ±9.1  ±11.3 | 0.1 ^b^  0.1 ^b^ | N/A  N/A | 0.63  0.39 |
| **Nephrological conditions** | **Total**  ***n*=27** | | **Reporters**  ***n*=12** | | **Non-reporters**  ***n*=15** | | **Effect size** | **(95% CI)** | ***p*-value** |
| Life satisfaction [range 1-10] | 7.8 | ±1.4 | 7.3 | ±1.6 | 8.3 | ±1.0 | 0.8 ^a^ | (>-0.1;1.6) | 0.05 |
| Self-rated health [range 1-4] | 2.3 | ±0.7 | 2.4 | ±0.8 | 2.2 | ±0.7 | -0.3 ^a^ | (-1.1;0.5) | 0.45 |
| Psychosomatic health [range 1-5] | 3.9 | ±0.9 | 3.8 | ±1.0 | 4.1 | ±0.8 | 0.3 ^a^ | (-0.5;1.1) | 0.43 |
| Health-related QoL, total [range 1-100]  Physical  Emotional  Social  School | 82.8  86.1  79.6  87.0  76.5 | ±15.7  ±17.6  ±18.3  ±17.0  ±20.8 | 80.0  85.8  76.4  81.4  73.2 | ±16.2  ±14.0  ±19.3  ±19.8  ±22.5 | 85.3  86.5  82.5  92.1  79.6 | ±15.5  ±21.0  ±17.8  ±12.9  ±19.6 | 0.3 ^a^  -0.2 ^b^  -0.2 ^b^  -0.3 ^b^  0.3 ^a^ | (-0.5;1.2)  N/A  N/A  N/A  (-0.5;1.1) | 0.43  0.49  0.38  0.12  0.47 |
| Internalizing symptoms [t-score] ^1^  Anxiety symptoms  Depressive symptoms | 37.2  39.7 | ±9.5  ±9.5 | 39.1  42.8 | ±9.4  ±11.9 | 35.7  37.2 | ±9.7  ±6.5 | 0.3 ^b^  0.3 ^b^ | N/A  N/A | 0.13  0.14 |
| **General pediatric conditions** | **Total**  ***n*=574** | | **Reporters**  ***n*=151** | | **Non-reporters**  ***n*=423** | | **Effect size** | **(95% CI)** | ***p*-value** |
| Life satisfaction [range 1-10] | 6.3 | ±1.8 | 5.7 | ±1.9 | 6.6 | ±1.7 | 0.5 ^a^ | (0.3;0.7) | <0.001 |
| Self-rated health [range 1-4] | 3.2 | ±0.7 | 3.5 | ±0.6 | 3.1 | ±0.7 | -0.7 ^a^ | (-0.8;-0.5) | <0.001 |
| Psychosomatic health [range 1-5] | 2.9 | ±0.7 | 2.7 | ±0.7 | 2.9 | ±0.7 | 0.3 ^a^ | (0.1;0.5) | 0.003 |
| Health-related QoL, total [range 1-100]  Physical  Emotional  Social  School | 61.3  57.4  62.9  75.4  51.5 | ±15.4  ±22.3  ±20.6  ±16.7  ±19.2 | 54.3  47.2  59.2  68.6  46.4 | ±15.1  ±23.9  ±19.4  ±18.3  ±18.7 | 63.6  60.9  64.2  77.8  53.3 | ±14.7  ±20.6  ±20.9  ±15.5  ±19.1 | 0.6 ^a^  -0.2 ^b^  -0.1 ^b^  -0.2 ^b^  0.4 ^a^ | (0.4;0.8)  N/A  N/A  N/A  (0.1;0.6) | <0.001  <0.001  0.24  <0.001  0.001 |
| Internalizing symptoms [t-score] ^1^  Anxiety symptoms  Depressive symptoms | 42.1  55.6 | ±10.8  ±12.5 | 42.9  58.6 | ±11.5  ±12.0 | 41.8  54.5 | ±10.5  ±12.6 | >0.01 ^b^  0.2 ^b^ | N/A  N/A | 0.38  <0.001 |

Data are presented as *n* or mean ± standard deviation. N/A means not applicable. ^1^ Raw scores were converted to normative T-scores based on sex and age, where <65 = normal, 65-70 = borderline, >70 = clinical. ^a^ = Cohen’s d; ^b^ = *r*.
