# Supplementary material for: Chronic conditions and adolescents’ psychosocial wellbeing: the impact of self-reporting
Source: Eur J Pediatr. 2025 Dec 13;185(1):15. doi: 10.1007/s00431-025-06616-5 (PMC12701850; doi:10.1007/s00431-025-06616-5)
Supplement: Supplementary file 3 — (DOCX 112 KB) [file 431_2025_6616_MOESM3_ESM.docx]

**Supplement 3.** List of diagnoses in the general pediatric conditions group

|  | **1** | **2** | **3** | **4** | **5** | **6** | **7** |
| --- | --- | --- | --- | --- | --- | --- | --- |
|  | acne vulgaris | chronic fatigue | functional abdominal pain | behçet’s disease | |  |  |
|  | deviating growth (height) | social anxiety | fatigue | |  |  |  |
|  | cachexia | chronic abdominal pain | cow milk’s protein allergy | constipation |  |  |  |
|  | chronic abdominal pain | diabetes mellitus | mandibular dysfunction | |  |  |  |
|  | chronic abdominal pain | |  |  |  |  |  |
|  | chronic fatigue | scoliosis | hip dysplasia | ibs |  |  |  |
|  | chronic fatigue | scoliosis | hypermobility | pectus carinatum |  |  |  |
|  | fibromyalgia | syncope | obesity |  |  |  |  |
|  | iron deficiency | adhd |  |  |  |  |  |
|  | chronic abdominal pain |  |  |  |  |  |  |
|  | chronic abdominal pain | constipation |  |  |  |  |  |
|  | chronic abdominal pain | constipation |  |  |  |  |  |
|  | chronic fatigue | hypothyroidy | |  |  |  |  |
|  | chronic pain | coccygodynia |  |  |  |  |  |
|  | chronic pain | herniated disc |  |  |  |  |  |
|  | constipation |  |  |  |  |  |  |
|  | diabetes type 1 | |  |  |  |  |  |
|  | avnrt | neuropathic pain | |  |  |  |  |
|  | orthostatic hypotension | mood disorder | |  |  |  |  |
|  | asthma | chronic pain | |  |  |  |  |
|  | chronic lung disease | migraine |  |  |  |  |  |
|  | chronic abdominal pain | precocious puberty | |  |  |  |  |
|  | coeliac disease |  |  |  |  |  |  |
|  | ulcerative colitis | juvenile idiopathic artritis | | |  |  |  |
|  | crohn’s disease | obesity |  |  |  |  |  |
|  | gastro-intestinal reflux disease | ibs |  |  |  |  |  |
|  | gastroparesis | asd |  |  |  |  |  |
|  | meckel's diverticulum | |  |  |  |  |  |
|  | crohn’s disease | |  |  |  |  |  |
|  | dysmenorrhoea | mood & anxiety disorder | | |  |  |  |
|  | acnes |  |  |  |  |  |  |
|  | autonomic dysfunction | migraine |  |  |  |  |  |
|  | cprs |  |  |  |  |  |  |
|  | epilepsy | gilbert's syndrome | |  |  |  |  |
|  | hemicrania continua | |  |  |  |  |  |
|  | right infrapatellar neuralgia | | |  |  |  |  |
|  | spondylolysis at l5 | |  |  |  |  |  |
|  | dermatofibrosarcoma protuberans | neuropathic pain | |  |  |  |  |
|  | arfid |  |  |  |  |  |  |
|  | autism | sinus bradycardia | |  |  |  |  |
|  | conversion disorder | |  |  |  |  |  |
|  | conversion disorder | |  |  |  |  |  |
|  | dyslexia | mood disorder | |  |  |  |  |
|  | mood disorder | obesity |  |  |  |  |  |
|  | jia | chronic fatigue | |  |  |  |  |
|  | takayasu arteritis |  |  |  |  |  |  |
|  | henoch schonlein vasculitis |  | |  |  |  |  |
|  | chronic pain | mood disorder | |  |  |  |  |
|  | chronic pain | |  |  |  |  |  |
|  | chronic pain | |  |  |  |  |  |
|  | chronic pain | |  |  |  |  |  |
|  | crps |  |  |  |  |  |  |
|  | neuralgia |  |  |  |  |  |  |
|  | neuropathy n.auricularis | hemimandible hyperplasia | | |  |  |  |
|  | nociceptive pain | |  |  |  |  |  |
|  | arthrogryposis multiplex congenita | chronic pain | |  |  |  |  |
|  | chronic pain | |  |  |  |  |  |
|  | chronic pain | hypermobility | |  |  |  |  |
|  | chronic pain | |  |  |  |  |  |
|  | tietze syndrome | |  |  |  |  |  |
|  | abdominal migraine | cachexia |  |  |  |  |  |
|  | respiratory dysregulation | chronic abdominal pain | |  |  |  |  |
|  | amplified pain syndrome | chronic fatigue | |  |  |  |  |
|  | chronic pain syndrome | |  |  |  |  |  |
|  | chronic pain syndrome | |  |  |  |  |  |
|  | chronic pain syndrome | |  |  |  |  |  |
|  | chronic pain syndrome | |  |  |  |  |  |
|  | chronic abdominal pain | |  |  |  |  |  |
|  | chronic abdominal pain | |  |  |  |  |  |
|  | chronic abdominal pain | chronic fatigue | |  |  |  |  |
|  | chronic headache | |  |  |  |  |  |
|  | chronic headache | mups, other |  |  |  |  |  |
|  | chronic headache | chronic fatigue | |  |  |  |  |
|  | chronic headache | chronic musculoskeletal pain | | | |  |  |
|  | chronic headache | chronic fatigue | chronic musculoskeletal pain | | | |  |
|  | chronic headache | mups, other |  |  |  |  |  |
|  | chronic headache | chronic abdominal pain | |  |  |  |  |
|  | chronic headache | chronic fatigue | |  |  |  |  |
|  | chronic headache | |  |  |  |  |  |
|  | chronic headache | chronic fatigue | |  |  |  |  |
|  | chronic headache | |  |  |  |  |  |
|  | chronic fatigue | |  |  |  |  |  |
|  | chronic fatigue | |  |  |  |  |  |
|  | chronic fatigue | functional abdominal pain | |  |  |  |  |
|  | chronic fatigue | |  |  |  |  |  |
|  | chronic fatigue | |  |  |  |  |  |
|  | chronic fatigue | |  |  |  |  |  |
|  | chronic fatigue | |  |  |  |  |  |
|  | chronic fatigue | |  |  |  |  |  |
|  | chronic fatigue | |  |  |  |  |  |
|  | chronic fatigue | chronic musculoskeletal pain | | | |  |  |
|  | chronic fatigue | |  |  |  |  |  |
|  | chronic fatigue | |  |  |  |  |  |
|  | chronic fatigue | dysfunctional breathing | | |  |  |  |
|  | chronic fatigue | functional dyspepsia | |  |  |  |  |
|  | chronic fatigue | |  |  |  |  |  |
|  | chronic fatigue | |  |  |  |  |  |
|  | chronic fatigue | chronic musculoskeletal pain | | | |  |  |
|  | chronic fatigue | |  |  |  |  |  |
|  | chronic fatigue | |  |  |  |  |  |
|  | chronic fatigue | |  |  |  |  |  |
|  | chronic fatigue | |  |  |  |  |  |
|  | chronic fatigue | |  |  |  |  |  |
|  | chronic fatigue | |  |  |  |  |  |
|  | chronic fatigue | |  |  |  |  |  |
|  | chronic fatigue | |  |  |  |  |  |
|  | chronic fatigue | chronic musculoskeletal pain | | | |  |  |
|  | chronic fatigue | chronic headache | |  |  |  |  |
|  | chronic fatigue | |  |  |  |  |  |
|  | chronic fatigue | |  |  |  |  |  |
|  | chronic fatigue | |  |  |  |  |  |
|  | chronic fatigue | |  |  |  |  |  |
|  | chronic fatigue | |  |  |  |  |  |
|  | chronic fatigue | chronic musculoskeletal pain | | | |  |  |
|  | chronic fatigue | |  |  |  |  |  |
|  | chronic fatigue | functional abdominal pain | |  |  |  |  |
|  | chronic fatigue | |  |  |  |  |  |
|  | chronic fatigue | |  |  |  |  |  |
|  | chronic fatigue | mups, other |  |  |  |  |  |
|  | chronic fatigue | |  |  |  |  |  |
|  | chronic fatigue | |  |  |  |  |  |
|  | chronic fatigue | chronic headache | |  |  |  |  |
|  | chronic fatigue | dysfunctional breathing | |  |  |  |  |
|  | chronic fatigue | functional dyspepsia | |  |  |  |  |
|  | chronic fatigue | mups, other |  |  |  |  |  |
|  | chronic fatigue | intellectual disability | mups, other |  |  |  |  |
|  | chronic fatigue | |  |  |  |  |  |
|  | chronic fatigue | chronic musculoskeletal pain | | | |  |  |
|  | chronic fatigue | |  |  |  |  |  |
|  | chronic fatigue | ibs |  |  |  |  |  |
|  | chronic fatigue | |  |  |  |  |  |
|  | chronic fatigue | hyperventilation | |  |  |  |  |
|  | chronic fatigue | mups, other |  |  |  |  |  |
|  | chronic fatigue | |  |  |  |  |  |
|  | chronic fatigue | chronic pain | |  |  |  |  |
|  | chronic fatigue | |  |  |  |  |  |
|  | chronic otalgia | |  |  |  |  |  |
|  | chronic pain | |  |  |  |  |  |
|  | chronic pain | ibs |  |  |  |  |  |
|  | chronic pain | hyperventilation syndrome | |  |  |  |  |
|  | chronic pain | |  |  |  |  |  |
|  | chronic pain | |  |  |  |  |  |
|  | chronic pain | |  |  |  |  |  |
|  | chronic pain | chronic fatigue | |  |  |  |  |
|  | chronic pain | |  |  |  |  |  |
|  | chronic musculoskeletal pain | | | |  |  |  |
|  | chronic musculoskeletal pain | | | |  |  |  |
|  | chronic fatigue syndrome |  |  |  |  |  |  |
|  | chronic fatigue syndrome | ibs |  |  |  |  |  |
|  | chronic fatigue syndrome |  |  |  |  |  |  |
|  | chronic fatigue syndrome |  |  |  |  |  |  |
|  | chronic fatigue syndrome |  |  |  |  |  |  |
|  | chronic fatigue syndrome |  |  |  |  |  |  |
|  | chronic fatigue syndrome |  |  |  |  |  |  |
|  | chronic fatigue syndrome |  |  |  |  |  |  |
|  | chronic fatigue syndrome |  |  |  |  |  |  |
|  | chronic fatigue syndrome | ibs |  |  |  |  |  |
|  | chronic fatigue syndrome |  |  |  |  |  |  |
|  | chronic fatigue syndrome | ibs |  |  |  |  |  |
|  | chronic fatigue syndrome |  |  |  |  |  |  |
|  | chronic fatigue syndrome |  |  |  |  |  |  |
|  | chronic fatigue syndrome |  |  |  |  |  |  |
|  | chronic fatigue syndrome |  |  |  |  |  |  |
|  | chronic fatigue syndrome |  |  |  |  |  |  |
|  | chronic fatigue syndrome | ibs |  |  |  |  |  |
|  | chronic fatigue syndrome |  |  |  |  |  |  |
|  | chronic fatigue syndrome | ibs |  |  |  |  |  |
|  | chronic fatigue syndrome |  |  |  |  |  |  |
|  | chronic fatigue syndrome |  |  |  |  |  |  |
|  | chronic fatigue syndrome |  |  |  |  |  |  |
|  | chronic fatigue syndrome | ibs |  |  |  |  |  |
|  | chronic fatigue syndrome |  |  |  |  |  |  |
|  | chronic fatigue syndrome |  |  |  |  |  |  |
|  | chronic fatigue syndrome |  |  |  |  |  |  |
|  | chronic fatigue syndrome |  |  |  |  |  |  |
|  | chronic fatigue syndrome |  |  |  |  |  |  |
|  | chronic fatigue syndrome |  |  |  |  |  |  |
|  | chronic fatigue syndrome |  |  |  |  |  |  |
|  | chronic fatigue syndrome |  |  |  |  |  |  |
|  | chronic fatigue syndrome |  |  |  |  |  |  |
|  | chronic fatigue syndrome |  |  |  |  |  |  |
|  | chronic fatigue syndrome |  |  |  |  |  |  |
|  | chronic fatigue syndrome |  |  |  |  |  |  |
|  | chronic fatigue syndrome |  |  |  |  |  |  |
|  | chronic fatigue syndrome |  |  |  |  |  |  |
|  | chronic fatigue syndrome |  |  |  |  |  |  |
|  | chronic fatigue syndrome |  |  |  |  |  |  |
|  | chronic fatigue syndrome |  |  |  |  |  |  |
|  | chronic fatigue syndrome |  |  |  |  |  |  |
|  | chronic fatigue syndrome |  |  |  |  |  |  |
|  | chronic fatigue syndrome |  |  |  |  |  |  |
|  | chronic fatigue syndrome |  |  |  |  |  |  |
|  | chronic fatigue syndrome |  |  |  |  |  |  |
|  | chronic fatigue syndrome |  |  |  |  |  |  |
|  | chronic fatigue syndrome |  |  |  |  |  |  |
|  | chronic fatigue syndrome |  |  |  |  |  |  |
|  | chronic fatigue syndrome |  |  |  |  |  |  |
|  | chronic fatigue syndrome |  |  |  |  |  |  |
|  | chronic fatigue syndrome |  |  |  |  |  |  |
|  | chronic fatigue syndrome |  |  |  |  |  |  |
|  | chronic fatigue syndrome |  |  |  |  |  |  |
|  | chronic fatigue syndrome | ibs |  |  |  |  |  |
|  | chronic fatigue syndrome |  |  |  |  |  |  |
|  | chronic fatigue syndrome |  |  |  |  |  |  |
|  | chronic fatigue syndrome |  |  |  |  |  |  |
|  | chronic fatigue syndrome |  |  |  |  |  |  |
|  | chronic fatigue syndrome |  |  |  |  |  |  |
|  | chronic fatigue syndrome |  |  |  |  |  |  |
|  | chronic fatigue syndrome |  |  |  |  |  |  |
|  | chronic fatigue syndrome |  |  |  |  |  |  |
|  | chronic fatigue syndrome |  |  |  |  |  |  |
|  | chronic fatigue syndrome |  |  |  |  |  |  |
|  | chronic fatigue syndrome |  |  |  |  |  |  |
|  | chronic fatigue syndrome |  |  |  |  |  |  |
|  | chronic fatigue syndrome |  |  |  |  |  |  |
|  | chronic fatigue syndrome | ibs |  |  |  |  |  |
|  | chronic fatigue syndrome |  |  |  |  |  |  |
|  | chronic fatigue syndrome |  |  |  |  |  |  |
|  | chronic fatigue syndrome |  |  |  |  |  |  |
|  | chronic fatigue syndrome |  |  |  |  |  |  |
|  | chronic fatigue syndrome |  |  |  |  |  |  |
|  | chronic fatigue syndrome |  |  |  |  |  |  |
|  | chronic fatigue syndrome |  |  |  |  |  |  |
|  | chronic fatigue syndrome |  |  |  |  |  |  |
|  | chronic fatigue syndrome |  |  |  |  |  |  |
|  | chronic fatigue syndrome |  |  |  |  |  |  |
|  | dysphagia |  |  |  |  |  |  |
|  | dysfunctional breathing | |  |  |  |  |  |
|  | fibromyalgia |  |  |  |  |  |  |
|  | fibromyalgia |  |  |  |  |  |  |
|  | fibromyalgia |  |  |  |  |  |  |
|  | functional abdominal pain | |  |  |  |  |  |
|  | functional abdominal pain | |  |  |  |  |  |
|  | functional abdominal pain | |  |  |  |  |  |
|  | functional abdominal pain | |  |  |  |  |  |
|  | functional abdominal pain | chronic fatigue syndrome |  |  |  |  |  |
|  | functional abdominal pain | |  |  |  |  |  |
|  | functional abdominal pain | |  |  |  |  |  |
|  | functional abdominal pain | |  |  |  |  |  |
|  | functional dyspepsia | |  |  |  |  |  |
|  | functional dyspepsia | |  |  |  |  |  |
|  | functional dyspepsia | ibs |  |  |  |  |  |
|  | functional dyspepsia | |  |  |  |  |  |
|  | functional gait disorder | |  |  |  |  |  |
|  | functional sensory disorder | | |  |  |  |  |
|  | functional fainting | |  |  |  |  |  |
|  | hyperventilation | |  |  |  |  |  |
|  | ibs |  |  |  |  |  |  |
|  | ibs |  |  |  |  |  |  |
|  | ibs |  |  |  |  |  |  |
|  | ibs |  |  |  |  |  |  |
|  | ibs |  |  |  |  |  |  |
|  | ibs |  |  |  |  |  |  |
|  | ibs |  |  |  |  |  |  |
|  | ibs |  |  |  |  |  |  |
|  | ibs |  |  |  |  |  |  |
|  | ibs |  |  |  |  |  |  |
|  | mups, other | chronic fatigue | ibs |  |  |  |  |
|  | mups, other |  |  |  |  |  |  |
|  | mups, other |  |  |  |  |  |  |
|  | mups, other |  |  |  |  |  |  |
|  | mups, other |  |  |  |  |  |  |
|  | mups, other |  |  |  |  |  |  |
|  | mups, other |  |  |  |  |  |  |
|  | mups, other |  |  |  |  |  |  |
|  | mups, other |  |  |  |  |  |  |
|  | mups, other |  |  |  |  |  |  |
|  | chronic pain syndrome | hyperhidrosis | jia |  |  |  |  |
|  | chronic pain syndrome | chronic fatigue | induced endometrial atrophy | | |  |  |
|  | chronic pain syndrome | tourette syndrome | |  |  |  |  |
|  | chronic abdominal pain | speech disorder | |  |  |  |  |
|  | chronic abdominal pain | chronic fatigue | chronic constipation | pdd nos | tinu syndrome | |  |
|  | chronic abdominal pain | urachal fistula |  |  |  |  |  |
|  | chronic abdominal pain | obesity |  |  |  |  |  |
|  | chronic abdominal pain | lactose intolerance | mild conductive hearing loss | motor development delay | constipation |  |  |
|  | chronic headache | chronic renal failure | perceptive hearing loss | | |  |  |
|  | chronic headache | obesity |  |  |  |  |  |
|  | chronic headache | chronic fatigue | eczema | henoch schonlein vasculitis | ibd | intracranial hypertension | spondylarthritis |
|  | chronic headache | chronic fatigue | chronic musculoskeletal pain | discopathy | osteochondrosis | |  |
|  | chronic headache | scoliosis |  |  |  |  |  |
|  | chronic headache | chronic fatigue | dyslexia |  |  |  |  |
|  | chronic headache | heterozygote beta-thalassemia | | |  |  |  |
|  | chronic headache | chronic musculoskeletal pain | intellectual disability | |  |  |  |
|  | chronic headache | chronic fatigue | mild erythrocytosis | |  |  |  |
|  | chronic fatigue | juvenile dermatomyositis | | |  |  |  |
|  | chronic fatigue | migraine |  |  |  |  |  |
|  | chronic fatigue | mast cell activation syndrome | congenital giant nevus | |  |  |  |
|  | chronic fatigue | anxiety disorder | chronic pain | autonomic dysregulation | |  |  |
|  | chronic fatigue | obesity | ptosis | migraine |  |  |  |
|  | chronic fatigue | anxiety disorder |  |  |  |  |  |
|  | chronic fatigue | diabetes mellitus type 1 | food allergy | |  |  |  |
|  | chronic fatigue | chronic musculoskeletal pain | epilepsy | functional abdominal pain | hypermobility | |  |
|  | chronic fatigue | adhd | idiopathic facial paralysis | |  |  |  |
|  | chronic fatigue | chronic pain | encephalopathy | |  |  |  |
|  | chronic fatigue | tinnitus |  |  |  |  |  |
|  | chronic fatigue | 3^rd^ degree av block | |  |  |  |  |
|  | chronic fatigue | chronic pain | lyme’s disease | |  |  |  |
|  | chronic fatigue | chronic pancreatitis | chronic musculoskeletal pain | ctrc-gene anomaly | functional abdominal pain | jia |  |
|  | chronic fatigue | single kidney | constipation | urge-incontinence | |  |  |
|  | chronic fatigue | eczema | developmental delay | |  |  |  |
|  | chronic fatigue | congenital myasthenic syndrome | ptosis | precocious puberty | |  |  |
|  | chronic fatigue | intellectual disability | |  |  |  |  |
|  | chronic fatigue | chronic musculoskeletal pain | ibd |  |  |  |  |
|  | chronic fatigue | adhd |  |  |  |  |  |
|  | chronic fatigue | complex regional pain syndrome | functional abdominal pain | |  |  |  |
|  | chronic fatigue | allergic asthma | migraine |  |  |  |  |
|  | chronic fatigue | familial tall stature | | |  |  |  |
|  | chronic fatigue | migraine |  |  |  |  |  |
|  | chronic fatigue | chronic spontaneous urticaria | | |  |  |  |
|  | chronic fatigue | incontinence | constipation | recurrent urinary tract infections | |  |  |
|  | chronic fatigue | ibs | sleep disorder |  |  |  |  |
|  | chronic fatigue | deviating growth (height) | congenital hip dysplasia | delayed sleep phase syndrome |  | | |
|  | chronic fatigue | eczema | familial hypercholesterolemia | | |  |  |
|  | chronic fatigue | crohn’s disease | polyarticulaire jia | |  |  |  |
|  | chronic fatigue | cardiac arrhythmia | secondary adrenal insufficiency | | |  |  |
|  | chronic fatigue | jia |  |  |  |  |  |
|  | chronic fatigue | gilbert's syndrome | von willebrand disease | |  |  |  |
|  | chronic fatigue | hashimoto hypothyroidy | vesicoureteral reflux | |  |  |  |
|  | chronic fatigue | chronic headache | asthma |  |  |  |  |
|  | chronic fatigue | mood disorder | |  |  |  |  |
|  | chronic fatigue | panuveitis |  |  |  |  |  |
|  | chronic fatigue | meckel's diverticum | ibs |  |  |  |  |
|  | chronic fatigue | eczema | tourette’s syndrome | |  |  |  |
|  | chronic fatigue | tendosynovitis | |  |  |  |  |
|  | chronic fatigue | chronic musculoskeletal pain | coeliac disease | ulcerative colitis | |  |  |
|  | chronic fatigue | juvenile myoclonus epilepsy | mood disorder | |  |  |  |
|  | chronic fatigue | eczema | asthma | ibs |  |  |  |
|  | chronic fatigue | duplicated collecting system kidney | constipation | mood disorder | gilbert’s syndrome | |  |
|  | chronic fatigue | mitral valve insufficiency | persisting ductus | speech and language developmental delay | | |  |
|  | chronic fatigue | secondary amenorrhoea | |  |  |  |  |
|  | chronic fatigue | chronic pain | mood disorder | |  |  |  |
|  | chronic fatigue | asthma |  |  |  |  |  |
|  | chronic fatigue | wpw syndrome | |  |  |  |  |
|  | chronic fatigue | uveitis intermedius/posterior | | |  |  |  |
|  | chronic fatigue | migraine | mups, other |  |  |  |  |
|  | chronic fatigue | eczema | asthma |  |  |  |  |
|  | chronic fatigue | eczema | asthma |  |  |  |  |
|  | chronic fatigue | scoliosis |  |  |  |  |  |
|  | chronic fatigue | migraine | raynaud’s | |  |  |  |
|  | chronic fatigue | sleep disorder |  |  |  |  |  |
|  | chronic fatigue | chronic pain | |  |  |  |  |
|  | chronic fatigue | hypermobility | eczema | epilepsy |  |  |  |
|  | chronic fatigue | coats disease | dysfunctional breathing | |  |  |  |
|  | chronic fatigue | obesity |  |  |  |  |  |
|  | chronic fatigue | distal arthrogryposis | |  |  |  |  |
|  | chronic fatigue | jia |  |  |  |  |  |
|  | chronic fatigue | hip dysplasia | |  |  |  |  |
|  | chronic fatigue | eczema | enthesopathy | scoliosis |  |  |  |
|  | chronic fatigue | jia |  |  |  |  |  |
|  | chronic fatigue | sjogren’s syndrome | |  |  |  |  |
|  | chronic fatigue | eczema | mitral valve prolapse | |  |  |  |
|  | chronic fatigue | chronic tonsillitis | |  |  |  |  |
|  | chronic fatigue | crohn’s disease | |  |  |  |  |
|  | chronic fatigue | chronic abdominal pain | allergy | |  |  |  |
|  | chronic fatigue | mctd |  |  |  |  |  |
|  | chronic fatigue | eczema | ibd |  |  |  |  |
|  | chronic fatigue | dyslexia | asthma |  |  |  |  |
|  | chronic fatigue | obstructive uropathy | ibs |  |  |  |  |
|  | chronic fatigue | chronic musculoskeletal pain | ibs | scn5a-gene anomaly | mood disorder | |  |
|  | chronic fatigue | hypergonadotropic hypogonadism | | |  |  |  |
|  | chronic fatigue | depression | anxiety disorder | food allergy | |  |  |
|  | chronic fatigue | dyslexia | eczema |  |  |  |  |
|  | chronic fatigue | ehlers danlos syndrome | |  |  |  |  |
|  | chronic fatigue | depression | musculoskeletal pain | | |  |  |
|  | chronic fatigue | mctd |  |  |  |  |  |
|  | chronic fatigue | obesity |  |  |  |  |  |
|  | chronic fatigue | add |  |  |  |  |  |
|  | chronic pain | adhd | hypermobility | |  |  |  |
|  | chronic pain | hypermobility | |  |  |  |  |
|  | chronic pain | asthma |  |  |  |  |  |
|  | chronic pain | mood disorder | |  |  |  |  |
|  | chronic pain | fragile-x-syndrome | |  |  |  |  |
|  | chronic pain | constipation |  |  |  |  |  |
|  | chronic pain | dcd | adhd | anxiety disorder | dysthymia | |  |
|  | chronic pain | hypermobility | |  |  |  |  |
|  | chronic pain | chronic fatigue | add |  |  |  |  |
|  | chronic pain | pierre robin sequence | pyramidal tract syndrome | developmental delay | congenital clubfeet | hypermobility | |
|  | chronic musculoskeletal pain | hypermobility | thyroid adenoma | |  |  |  |
|  | chronic musculoskeletal pain | chronic fatigue syndrome | hypermobility | |  |  |  |
|  | chronic musculoskeletal pain | scoliosis | mups, other | gilbert syndrome | |  |  |
|  | chronic musculoskeletal pain | functional movement disorder | functional cognitive disorder | acquired brain injury | scoliosis |  |  |
|  | chronic fatigue syndrome | hypermobility | |  |  |  |  |
|  | chronic fatigue syndrome | eczema |  |  |  |  |  |
|  | chronic fatigue syndrome | hypermobility | |  |  |  |  |
|  | chronic fatigue syndrome | inguinale testis | migraine | pdd nos | atopic eczema | chronic abdominal pain | |
|  | chronic fatigue syndrome | fibromyalgia | scoliosis |  |  |  |  |
|  | chronic fatigue syndrome | deep sleep parasomnia | |  |  |  |  |
|  | chronic fatigue syndrome | mitral valve prolapse | |  |  |  |  |
|  | chronic fatigue syndrome | iron deficiency anaemia | eczema |  |  |  |  |
|  | chronic fatigue syndrome | chronic abdominal pain | |  |  |  |  |
|  | chronic fatigue syndrome | constipation |  |  |  |  |  |
|  | chronic fatigue syndrome | patellofemoral pain syndrome | | |  |  |  |
|  | chronic fatigue syndrome | hashimoto | |  |  |  |  |
|  | chronic fatigue syndrome | henoch schonlein vasculitis | |  |  |  |  |
|  | chronic fatigue syndrome | asthma |  |  |  |  |  |
|  | chronic fatigue syndrome | atopic eczema | |  |  |  |  |
|  | chronic fatigue syndrome | constipation | hypothyroidy | |  |  |  |
|  | chronic fatigue syndrome | dyslexia |  |  |  |  |  |
|  | chronic fatigue syndrome | adhd | hypermobility |  | |  |  |
|  | chronic fatigue syndrome | hyperventilation syndrome | migraine | von willebrand disease | |  |  |
|  | chronic fatigue syndrome | juvenile idiopathic artritis | | |  |  |  |
|  | chronic fatigue syndrome | chronic pain | asthma | tietze syndrome | |  |  |
|  | chronic fatigue syndrome | chronic pain | |  |  |  |  |
|  | chronic fatigue syndrome | heavy menstrual bleeding | | |  |  |  |
|  | chronic fatigue syndrome | ibs | mups, other | ichthyosis | eczema |  |  |
|  | chronic fatigue syndrome | eczema |  |  |  |  |  |
|  | chronic fatigue syndrome | headache |  |  |  |  |  |
|  | chronic fatigue syndrome | ichtyosis |  |  |  |  |  |
|  | chronic fatigue syndrome | scoliosis | dyslexia |  |  |  |  |
|  | chronic fatigue syndrome | eczema | hashimoto |  |  |  |  |
|  | chronic fatigue syndrome | constipation |  |  |  |  |  |
|  | chronic fatigue syndrome | congenital primary hypothyroidy | | |  |  |  |
|  | chronic fatigue syndrome | ibs | lyme’s disease | |  |  |  |
|  | chronic fatigue syndrome | post-concussion syndrome | |  |  |  |  |
|  | chronic fatigue syndrome | dyslexia |  |  |  |  |  |
|  | chronic fatigue syndrome | diabetes mellitus type 1 | |  |  |  |  |
|  | chronic fatigue syndrome | psychogenic insults | |  |  |  |  |
|  | chronic fatigue syndrome | hypertension |  |  |  |  |  |
|  | chronic fatigue syndrome | constipation |  |  |  |  |  |
|  | chronic fatigue syndrome | asthma |  |  |  |  |  |
|  | chronic fatigue syndrome | eczema | lumbosacral radicular syndrome | | |  |  |
|  | chronic fatigue syndrome | eating disorder | anxiety disorder |  |  |  |  |
|  | chronic fatigue syndrome | pathologically enlarged bladder | |  |  |  |  |
|  | chronic fatigue syndrome | mood disorder | |  |  |  |  |
|  | chronic fatigue syndrome | jia |  |  |  |  |  |
|  | chronic fatigue syndrome | lumbosacral meningomyelocele | neurogenic bladder | neurogenic intestinal function problems | | |  |
|  | chronic fatigue syndrome | dyslexia |  |  |  |  |  |
|  | chronic fatigue syndrome | food allergy | ibs |  |  |  |  |
|  | chronic fatigue syndrome | food allergy | |  |  |  |  |
|  | dysfunctional breathing | asthma | chronic fatigue syndrome |  |  |  |  |
|  | dysfunctional breathing | functional abdominal pain | allergic asthma | allergic rhinitis | |  |  |
|  | dysfunctional breathing | constipation | eczema |  |  |  |  |
|  | fibromyalgia | ibs | scoliosis |  |  |  |  |
|  | fibromyalgia | grave’s disease | |  |  |  |  |
|  | functional abdominal pain | food allergy | |  |  |  |  |
|  | functional abdominal pain | precocious puberty | |  |  |  |  |
|  | functional abdominal pain | iron deficiency |  |  |  |  |  |
|  | functional abdominal pain | ptsd |  |  |  |  |  |
|  | hyperventilation | migraine |  |  |  |  |  |
|  | hyperventilation | hypertensive kidney disease | |  |  |  |  |
|  | ibs | delayed sleep phase syndrome | | |  |  |  |
|  | ibs | scoliosis |  |  |  |  |  |
|  | ibs | atopic eczema | |  |  |  |  |
|  | ibs | conversion | |  |  |  |  |
|  | mups, other | constipation | cow’s milk protein allergy | |  |  |  |
|  | mups, other | depression | atopic asthma | |  |  |  |
|  | mups, other | atopic eczema | anosmia | pfapa-syndrome | |  |  |
|  | mups, other | adhd |  |  |  |  |  |
|  | mups, other | tinnitus | migraine |  |  |  |  |
|  | mups, other | coeliac disease | perniosis |  |  |  |  |
|  | mups, other | autism |  |  |  |  |  |
|  | mups, other | vestibular paroxysm | |  |  |  |  |
|  | mups, other | pdd-nos | adhd | dyslexia |  |  |  |
|  | chronic headache | functional abdominal pain | chronic fatigue | |  |  |  |
|  | chronic fatigue | hypothyroidy | systemic lupus erythematodes |  |  |  |  |
|  | chronic fatigue | purine nucleoside phosporylase deficiency | developmental disorder | autism |  |  |  |
|  | chronic fatigue | asthma | sleep disorder |  |  |  |  |
|  | constipation | pelvic floor hypertonia | tension-type headache | chronic fatigue syndrome | mups, other |  |  |
|  | constipation | hypermenorrhoea | mood disorder | |  |  |  |
|  | constipation | arfid | primary adrenal insufficiency | graves disease | dysfunctional voiding | |  |
|  | tension-type headache | diabetes mellitus | chronic pain | |  |  |  |
|  | chronic rhinitis | functional abdominal pain | constipation |  |  |  |  |
|  | diabetes mellitus | mups, other |  |  |  |  |  |
|  | avnrt | chronic fatigue | |  |  |  |  |
|  | bicuspid aorta valve | chronic fatigue | migraine |  |  |  |  |
|  | essential hypertension | mups, other |  |  |  |  |  |
|  | bone marrow dysplasia | chronic fatigue | coeliac disease |  |  |  |  |
|  | eczema | ibs | mups, other |  |  |  |  |
|  | allergic asthma | chronic fatigue | hypermobility | constipation | secondary nocturnal enuresis | | |
|  | allogeneic lung pattern | mood & anxiety disorders | chronic fatigue | graft vs host disease | migraine | paroxysmal haemoglobinuria | |
|  | asthma | functional abdominal pain | |  |  |  |  |
|  | asthma | chronic fatigue | chronic musculoskeletal pain | obesity |  |  |  |
|  | asthma | chronic headache | chronic fatigue | |  |  |  |
|  | asthma | conversion | depression | constipation |  |  |  |
|  | asthma | chronic headache | chronic fatigue syndrome | eczema |  |  |  |
|  | asthma | chronic fatigue | constipation | ibs |  |  |  |
|  | asthma | chronic fatigue | eczema | functional abdominal pain | sickle cell trait |  |  |
|  | asthma | chronic headache | chronic fatigue | epilepsy |  |  |  |
|  | asthma | chronic fatigue | |  |  |  |  |
|  | asthma | chronic recurrent multifocal osteomyelitis | chronic fatigue syndrome |  |  |  |  |
|  | asthma | chronic fatigue | coeliac disease | dysimmunoglobulinemia | eczema | mups, other |  |
|  | asthma | chronic fatigue | gilbert syndrome | |  |  |  |
|  | asthma | chronic otitis media | chronic musculoskeletal pain | eczema | multiple allergies | |  |
|  | asthma | chronic fatigue syndrome | hashimoto |  |  |  |  |
|  | asthma | dysfunctional breathing | lactose intolerance | |  |  |  |
|  | asthma | chronic fatigue | goldenhar syndrome | |  |  |  |
|  | asthma | chronic fatigue | |  |  |  |  |
|  | asthma | chronic musculoskeletal pain | insect allergy | adenoid hypertrophy | ibs |  |  |
|  | asthma | atopic eczema | migraine | food allergy | |  |  |
|  | asthma | chronic urticaria | chronic fatigue syndrome | ibs |  |  |  |
|  | asthma | chronic fatigue | chronic musculoskeletal pain | cvid | ibd |  |  |
|  | asthma | chronic fatigue | eczema | spinal muscle atrophy | |  |  |
|  | coeliac disease | ibs | chronic fatigue | |  |  |  |
|  | adpkd | hypertension | obesity | chronic pain | |  |  |
|  | congenital ureter pathology | chronic abdominal pain | recurrent urinary tract infections | |  |  |  |
|  | endometriosis | pelvic floor hypertonia | mups, other |  |  |  |  |
|  | acnes | chronic pain | |  |  |  |  |
|  | acnes | chronic fatigue | conversion | hashimoto | constipation |  |  |
|  | central sensitisation | chronic fatigue | |  |  |  |  |
|  | chiari malformation | chronic fatigue | chronic musculoskeletal pain | epilepsy | sensory processing disorder | |  |
|  | chiari malformation type 1 | headache | syringomyelia | |  |  |  |
|  | chronic fatigue | food allergy | nasal obstruction | deviating growth | |  |  |
|  | cluster headache | chronic fatigue syndrome |  |  |  |  |  |
|  | kaposiform hemangioendothelioma | neuroma of the sciatic nerve | neuropathic pain | right transfemoral amputation | | |  |
|  | add | chronic fatigue | eczema |  |  |  |  |
|  | add | asthma | chronic pain syndrome | eczema |  |  |  |
|  | add | chronic fatigue | |  |  |  |  |
|  | add | chronic fatigue | |  |  |  |  |
|  | add | chronic fatigue | dysfunctional voiding | constipation | mups, other |  |  |
|  | add | chronic fatigue | dyslexia | igg2 deficiency | |  |  |
|  | add | fibromyalgia | obesity |  |  |  |  |
|  | adhd | chronic fatigue | |  |  |  |  |
|  | adhd | chronic active hbv-infection | chronic fatigue | chronic otitis media | eczema |  |  |
|  | adhd | chronic fatigue | hypermobility | |  |  |  |
|  | adhd | asthma | chronic fatigue | migraine |  |  |  |
|  | adhd | chronic fatigue | cutanic lupus erythematosus | | |  |  |
|  | adhd | anxiety disorder | ibs |  |  |  |  |
|  | adhd | chronic fatigue | |  |  |  |  |
|  | adhd | chronic abdominal pain | |  |  |  |  |
|  | adhd | chronic fatigue | hashimoto | dysfunctional voiding | faecal incontinence | |  |
|  | adhd | chronic fatigue | dyslexia | jia |  |  |  |
|  | adhd | chronic pain syndrome | femoral anteversion | menstrual cycle disorder | dyscalculia | dyslexia |  |
|  | adhd/add | chronic fatigue | |  |  |  |  |
|  | adhd/add | mood & anxiety disorder | functional abdominal pain | |  |  |  |
|  | adhd/add | chronic fatigue | |  |  |  |  |
|  | adhd/add | asd | asthma | chronic fatigue | eczema | food allergy | |
|  | adhd/add | chronic fatigue syndrome |  |  |  |  |  |
|  | adhd/add | chronic fatigue | hypermobility | |  |  |  |
|  | adhd/add | mood & anxiety disorders | chronic fatigue syndrome |  |  |  |  |
|  | adhd/add | chronic headache | |  |  |  |  |
|  | adhd/add | asd | coeliac disease | chronic fatigue syndrome | hypothyroidy | |  |
|  | adhd/add | dysfunctional voiding | constipation | gastroparesis | |  |  |
|  | adhd/add | chronic headache | chronic fatigue | growth hormone deficiency | constipation |  |  |
|  | adhd/add | chronic headache | chronic fatigue syndrome | dyslexia | gastropareses | | |
|  | mood & anxiety disorder | chronic fatigue | trauma capitis | von willebrand disease | |  |  |
|  | mood & anxiety disorder | chronic headache | chronic fatigue | functional abdominal pain | |  |  |
|  | mood & anxiety disorder | chronic fatigue | |  |  |  |  |
|  | mood & anxiety disorder | chronic fatigue syndrome | obesity |  |  |  |  |
|  | mood & anxiety disorder | eczema | hyperhidrosis | ibs |  |  |  |
|  | mood & anxiety disorder | chronic fatigue | functional abdominal pain | hypertrichosis | |  |  |
|  | mood & anxiety disorder | ibs |  |  |  |  |  |
|  | mood & anxiety disorder | chronic fatigue | fibromyalgia |  |  |  |  |
|  | mood & anxiety disorder | asd | constipation |  |  |  |  |
|  | mood & anxiety disorder | chronic fatigue syndrome |  |  |  |  |  |
|  | mood & anxiety disorder | chronic headache | chronic fatigue | obesity |  |  |  |
|  | mood & anxiety disorders | chronic musculoskeletal pain | chronic fatigue syndrome | ibs |  |  |  |
|  | mood & anxiety disorders | asthma | chronic musculoskeletal pain | | | |  |
|  | mood & anxiety disorders | asthma | chronic fatigue | coeliac disease |  |  |  |
|  | anxiety disorder | chronic headache | tourette syndrome | partial epilepsy | |  |  |
|  | anxiety disorder | asd | chronic pain syndrome | obesity |  |  |  |
|  | anxiety disorder | chronic fatigue | |  |  |  |  |
|  | anxiety disorder | chronic dyspepsia | chronic fatigue | |  |  |  |
|  | anxiety disorder | chronic fatigue | |  |  |  |  |
|  | anorexia nervosa | chronic fatigue syndrome | dysfunctional breathing | migraine |  |  |  |
|  | mood & anxiety disorder | chronic fatigue syndrome |  |  |  |  |  |
|  | asd | chronic fatigue syndrome | functional abdominal pain | |  |  |  |
|  | asd | chronic fatigue | chronic musculoskeletal pain | | | |  |
|  | asd | chronic fatigue | obesity |  |  |  |  |
|  | asd | chronic fatigue | |  |  |  |  |
|  | asd | chronic fatigue | asthma | constipation | urge-incontinence | |  |
|  | asd | chronic fatigue | ptsd |  |  |  |  |
|  | asd | chronic fatigue | |  |  |  |  |
|  | asd | chronic musculoskeletal pain | functional abdominal pain | |  |  |  |
|  | asd | single kidney and shrunken kidney | musculoskeletal pain | | |  |  |
|  | autism | auto-immune hepatitis | chronic fatigue | dyslexia |  |  |  |
|  | conversion | chronic fatigue syndrome | ibs | multinodular struma | |  |  |
|  | conversion | graves disease | precocious puberty | |  |  |  |
|  | dysthymia | adhd | asthma |  |  |  |  |
|  | pdd nos | recurrent epididymitis | mups, other |  |  |  |  |
|  | acnes | constipation | hypermobility | |  |  |  |
|  | chronic pain | lymphangiomatosis | venous malformation | |  |  |  |
|  | pelvic floor hypertonia | chronic pain | oligo-amenorrhoea | |  |  |  |
|  | crps | foetal alcohol syndrome | ptsd |  |  |  |  |
|  | hypermobility | hyperventilation | |  |  |  |  |
